# Synthesis and in Vitro Cytocompatibility of Segmented Poly(Ester-Urethane)s and Poly(Ester-Urea-Urethane)s for Bone Tissue Engineering

**DOI:** 10.3390/polym10090991

**Published:** 2018-09-05

**Authors:** Dulce María González-García, Ángel Marcos-Fernández, Luis M. Rodríguez-Lorenzo, Rodrigo Jiménez-Gallegos, Nancy Vargas-Becerril, Lucía Téllez-Jurado

**Affiliations:** 1 Departamento de Ingeniería Metalúrgica, Instituto Politécnico Nacional, ESIQIE, UPALM-Zacatenco, Col Lindavista, México City 07738, Mexico; rodrigojg04@gmail.com (R.J.-G.); nancyvb09@gmail.com (N.V.-B.); ltellezj@ipn.mx (L.T.-J.); 2 Instituto de ciencia y tecnología de Polímeros, ICTP-CSIC calle Juan de la Cierva 3, 28006 Madrid, Spain; amarcos@ictp.csic.es (A.M.-F.); luis.rodriguez-lorenzo@ictp.csic.es (L.M.R.-L.); 3 CIBER-BBN, C. Monforte de Lemos 3-5, Pabellón 11, 28029 Madrid, Spain

**Keywords:** polyurethanes, biodegradable polymers, cytocompatibility, human osteoblastic cells

## Abstract

Two series of segmented polyurethanes were obtained and their mechanical and thermal properties as well as their biodegradability and cytotoxicity were evaluated. The chemical nature of the polyurethanes was varied by using either 1,4 butanediol (poly-ester-urethanes, PEUs) or l-lysine ethyl ester dihydrochloride (poly-ester-urea-urethanes, PEUUs) as chain extenders. Results showed that varying the hard segment influenced the thermal and mechanical properties of the obtained polymers. PEUs showed strain and hardness values of about 10–20 MPa and 10–65 MPa, respectively. These values were higher than the obtained values for the PEUUs due to the phase segregation and the higher crystallinity observed for the polyester-urethanes (PEUs); phase segregation was also observed and analyzed by XRD and DSC. Moreover, both series of polymers showed hydrolytic degradation when they were submerged in PBS until 90 days with 20% of weight loss. In vitro tests using a Human Osteoblastic cell line (Hob) showed an average of 80% of cell viability and good adhesion for both series of polymers.

## 1. Introduction.

Cartilage and bone are the most commonly transplanted tissue after blood and they are the two tissues most attempted to replicate after skin. However, the devices already studied do not have the necessary qualities for its application [1]. The main challenge in creating suitable devices includes the proper selection of materials and properties. For this reason, a wide range of new materials are being investigated including biodegradable synthetic polymers such as poly(α-esters), polymers derived from L-tyrosine, poly(propylene fumarate), and polyphosphazenes [2]. Polycaprolactone (PCL) is one of the most extensively studied biodegradable polyesters. The degradation product of PCL is 6-hydroxyhexanoic acid, which is naturally metabolized by the human body. The toxicology of polycaprolactone materials have been studied and mentioned as safe devices for several clinical treatments; also, injectable scaffold based crosslinked polycaprolactones have been probed for bone regeneration showing good biological properties [3,4,5]. However, elastomers such as polyurethanes offer some unique advantages in terms of mechanical flexibility, tissue-biocompatibility, and tunable biodegradability. As a result, polyurethanes have been the most studied polymer in the biomedical field [6,7].

Polyurethane-based cell-free scaffolds have been implanted to treat painful partial meniscus loss. An effective treatment in both cases, in isolated partial meniscal lesions and in complex cases requiring the combination with other surgical procedures, has been reported [8,9]. From the therapeutic production and commercial points of view, cell-free scaffolds constitute the most suitable systems for bone regeneration [10]. One of the great advantages of elastomeric polyurethane scaffolds is the intimate contact between the implant and the native bone. This contact helps to eliminate shear forces at the bone-implant interface and facilitate the proliferation of osteogenic cells, which promotes bone regeneration. Other advantages of polyurethanes is the possibility of designing them in order to obtain the desired properties, e.g., the use of aliphatic segments avoids the toxicity of the degradation products and the use of peptides or amino acids as chain extenders can include functional groups [2,11]. In addition, the hydrolytic/hydrophobic character of polyurethanes affects their cell viability [12].

Recently, the interest in the design of new biodegradable-segmented polyurethanes is increasing due to their possible application as an artificial extracellular matrix in tissue engineering. The correct selection of soft and hard segments and their molar ratio influences the physical, mechanical, and biological properties required for a specific application [13,14,15]. The incorporation of labile ester linkages in the soft segment by using polycaprolactones or polylactides improves the material degradation [16,17]. In addition, the chemical composition of the hard segments affects the mechanical and biological properties of the materials [18,19]. In biodegradable polyurethanes, the hard segments are commonly composed by selected aliphatic diisocyanates, which proved to render non-cytotoxic degradation products [20,21].

Only a few authors have described the use of amino acids as chain extenders [19,22,23,24,25] and there is a lack of works that have analyzed their influence on the chemical, mechanical, and biological properties [26]. Tatai et al. [27] obtained a series of poly-ester-urethanes introducing a degradable chain extender and found differences in the degradation rates, but did not find differences in the mechanical or thermal properties.

In this manuscript, a series of biodegradable poly(ester-urethane)s and poly(ester-urea-urethane)s were synthesized. The influence of the molecular weight of the soft segment, the type of chain extender used in the mechanical properties, the thermal and degradative behavior, and cytocompatibility were evaluated. Furthermore, the potential application of these materials in bone regeneration was studied.

## 2. Experimental

### 2.1. Materials

Polycaprolactone diols (PCLs) of 530 and 2000 g·mol^−1^ nominal weight, 1,6-diisocyanatohexane (HDI), 1,4-butanediol (BD), *N*,*N*-Dimethylacetamide (DMAc), tin (II) 2-ethylhexanoate (Sn(Oct)_2_), l-lysine ethyl ester dihydrochloride (LYS), and triethylamine (TEA) were supplied by Sigma-Aldrich S.L (Madrid, Spain). LYS was dried in vacuum and HDI and BD were distilled prior to reaction.

A polycaprolactone diol of approximately 1000 g·mol^−1^ was synthesized in the laboratory following the general procedure for the synthesis of polycaprolactones by the ring opening polymerization of ε-caprolactone using diethylene glycol as the initiator and Sn(Oct)_2_ as the catalyst [16].

PCLs were vacuum dried at 80 °C for 12 h and stored in a desiccator until used. The exact molecular weight of the polycaprolactones was measured by proton NMR (diagram not shown) after derivatization as described in the literature [25].

### 2.2. Synthesis of Polyurethanes

Poly(ester-urethane)s (PEUs) and poly(ester-urea-urethane)s (PEUUs) were synthesized by the prepolymer method. The *M*w of the PCL, PEUs, and PEUUs codes, and reactant ratios are shown in Table 1.

Poly(ester-urethane)s were prepared by the following procedure. First, the polycaprolactone diol was charged in a reaction flask with 1,6-diisocyanatohexane (HDI) and 1% mol with respect to the PCL moles of tin(II) 2-ethylhexanoate (Sn(Oct)_2_). The mixture underwent a reaction at 80 °C with magnetic stirring for 3 h. Later, 1,4 butanediol (BD) was added and the reaction was allowed to proceed at 80 °C for another 4 h. *N*,*N* Dimethylacetamide (DMac) was added before the chain extender was reacted, using a ratio of 50:50 (*w*/*v* weight of reactants in gram/volume of DMAc in mL).

Poly(ester-urea-urethane)s were obtained following the procedure described by Marcos et al. [25]. The polycaprolactone diols (PCLs 530, 1000, and 2000) were used as the soft segment and the appropriate amounts of HDI and L-lysine ethyl ester dihydrochloride (LYS) were used as the hard segment.

The reactant ratio of PCL:HDI: chain extender was varied in order to obtain a 30% weight of hard segment (defined as % hard segment = [weight of diisocianate + weight of chain extender]/total weight of monomers × 100) in all of the polymers synthesized.

### 2.3. Preparation of Polyurethane Films

The resulting polyurethanes were washed with deionized water and vacuum dried for 48 h. Films were obtained by solvent casting by dissolving 3 g of material in DMAc and casting on Teflon, followed by heating at 80 °C for 24 h.

### 2.4. Characterization Techniques

#### 2.4.1. Size Exclusion Chromatography (SEC)

The average molecular weights of the PEUs and PEUUs were determined by size exclusion chromatography (SEC) using a Perkin Elmer series 200 gel permeation chromatograph equipped with a refractive index detector. A set of 10^4^, 10^3^, and 100 Å Waters columns conditioned at 35 °C was used to elute samples at a 1 mL min^−1^ flow rate with HPLC-grade dimethylformamide with 1% of BrLi as the solvent. Polystyrene standards were used for the calibration.

#### 2.4.2. Proton Nuclear Magnetic Resonance (^1^H-NMR)

Proton Nuclear Magnetic Resonance (^1^H-NMR) spectra were used to calculate the molecular weight of the PCLs [23] by using a Varian 400 spectrometer (Varian, Palo Alto, CA, USA) and deuterated chloroform (CDCL_3_) as the solvent. The residual signal of the deuterated solvent was used as the internal reference (7.26 ppm).

#### 2.4.3. Infrared Spectroscopy (FT-IR)

The infrared spectra (FT-IR) were obtained using a Perkin-Elmer spectrometer coupled with an attenuated total reflection accessory (Perkin-Elmer, Billerica, MA, USA) at room temperature. The spectra were measured in the range of 4000 to 550 cm^−1^ using 16 scans with 2 cm^−1^ of resolution.

#### 2.4.4. Thermogravimetric Analysis (TGA)

The thermogravimetric analysis (TGA) of samples was carried out in a Mettler-Toledo TGA/SDTA 851 instrument (Mettler-Toledo, Schwerzenbach, Switzerland) from room temperature to 500 °C under a nitrogen atmosphere at a 10 °C/min heating rate.

#### 2.4.5. Differential Scanning Calorimetry (DSC)

The thermal transitions of the polymers were studied by differential scanning calorimetry (DSC) in a Perkin Elmer DSC-7 instrument (Perkin-Elmer, Billerica, MA, USA) with a heating rate of 10 °C/ min from −80 to 180 °C under a nitrogen atmosphere. The melting points (*M*_p_) were taken from the maximum of endothermic transitions, and glass transition temperatures (*T*_g_) were reported as the mid-point of transitions.

#### 2.4.6. X-ray Diffraction (XRD)

X-ray diffraction (XRD) were conducted using a Bruker D8 Advance (Bruker, Billerica, MA, USA) with Cu kα radiation at room temperature from 5 to 50° in 2θ at a 0.02° scan speed and 0.5 s per step.

#### 2.4.7. Mechanical Properties

Tensile properties were measured using a MTS Synergie 200 testing machine (MTS Systems, Eden Prairie, MN, USA) equipped with a 100 N load cell. Type 4 dumbbell test pieces (according to ISO 37) were cut from film. A cross head speed of 200 mm min^−1^ was used and strain was measured from the crosshead separation and referred to 12 mm initial length.

The Vickers hardness of the PEUs and PEUUs was tested at room temperature in a Leitz RZD-DO (Ernest Leitz GmbH, Wetzlar, Germany) apparatus with a load of 4.809 N. The indentation time was kept constant at 25 s and the results reported in MPa.

#### 2.4.8. Scanning Electron Microscopy (SEM)

Morphology and surface of the polymer films were observed in a scanning electron microscope, SEM (HITACHI, Tokyo, Japan) model HITACHI SU 800.

### 2.5. In Vitro Test

#### 2.5.1. Hydrolitic Degradation

The in vitro degradation of the polymers was evaluated by following the weight changes after the films were submerged in phosphate buffer solution (Sigma-Aldrich, Saint Louis, MO, USA) at 37 °C for 1, 3, 7, 14, 21, and 90 days. The water uptake percentage was taken from the maximum of the relation: weight change % = (*W*_m_ − *W*_o_/*W*_o_) × 100, where *W*_m_ is the weight of the hydrated specimen and *W*_o_ is the initial weight of the specimen. At least three specimens were tested for each polymer.

#### 2.5.2. Alamar Blue Test

The Alamar blue bioassay has been used to evaluate cytotoxicity and cell viability in biological systems, then shows the behavior of cells in contact with the surface of the polymers synthesized. Studies were performed using human osteoblast cells Hob, from (INNOPROT, P10979) with a density of 0.5 × 10^6^ cells. Cultures were maintained at 37 °C in humidified air with a 5% vol of CO_2_, and the culture medium was carefully replaced at selected time intervals under sterile conditions. The culture medium was Dulbeco’s modified Eagle’s Medium/HAM F12 enriched with 110 mg mL^−1^ of sodium pyruvate (DMEM, Thermo Fisher, Waltham, MA, USA) and supplemented with a 10% vol of fetal bovine serum (FBS, Gibco, Waltham, MA, USA), 200 mM of l-glutamine, 100 units mL^−1^ of penicillin, and 100 mg mL^−1^ of streptomycin (Sigma-Aldrich, Madrid, Spain). The cell adhesion was observed by SEM in a HITACHI SU 800 at 48 h and seven days of test.

#### 2.5.3. Statistical Analysis of Biocompatibility

A one-way ANOVA was performed by using STATA/SE software, Tukey post hoc test was used for pairwise comparisons of means with equal variances. The statistical analysis of Alamar blue was made with respect to the control, * *p* < 0.05.

## 3. Results

### 3.1. PEUs and PEUUs Synthesis

PEUs and PEUUs were synthesized using PCL diol as the soft segment. The molecular weights of the PCL diol selected were 530, 1000, and 2000 g·mol^−1^ and a 30% weight of hard segment was kept constant in all of the samples. The PEU and PEUU molecular weights increased from the PCL molecular weight used, as shown in Table 1. The increase of molecular weight in PEUU530 was lower than in any other PEU or PEUU synthesized. However, when compared to other biodegradable segmented polyurethanes found in the literature [25,28], the molecular weight was much higher for the polyurethanes prepared in this work.

**Figure 1:**
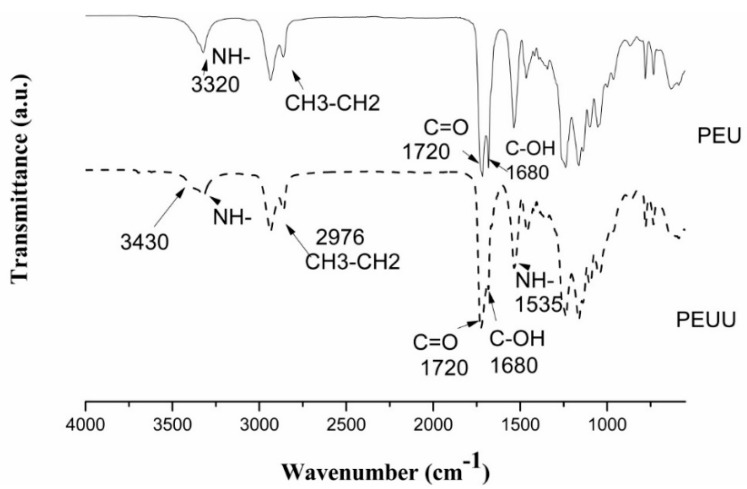
FT-IR spectra of PEUs and PEUUs.

As all of the spectra of PEUs and PEUUs were similar regardless of what molecular weight was used, a representative FT-IR spectra of the PEUs and PEUUs is shown in Figure 1. In the amine region, a broad peak corresponding to the N-H vibrations in the hydrogen bonded urea and urethane groups was observed at 3320 cm^−1^. A shoulder at 3430 cm^−1^ related to non-hydrogen bonded N–H groups was observed for PEUU but not for PEU, it can be related to the lysine used as a chain extender being difficult for the hard segment arrangement and promoted an asymmetric structure. Furthermore, the C=O absorption at 1720 cm^−1^ was related to the ester groups of the PCL soft segment and was observed for both spectra; moreover, (C-OH) non-hydrogen bonded carbonyl groups at 1680 cm^−1^ were found. 

**Figure 2:**
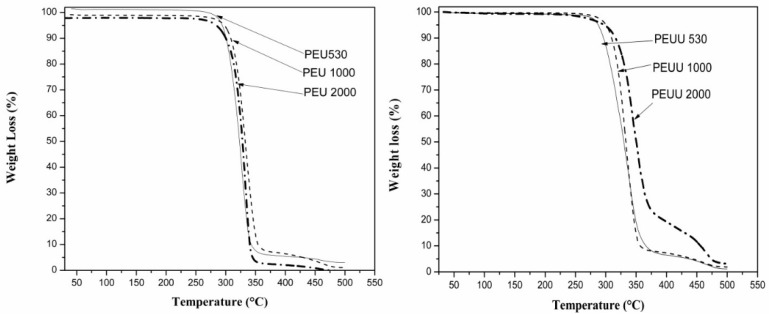
TGA curves of the synthesized polymers. (**Left**) PEUs, (**Right**) PEEUs.

Figure 2 shows the thermogravimetric curves for the PEUs and PEUUs. A single weight loss step was observed in all cases with degradation onset at 300 °C or above. Thus, these materials are relatively stable to temperature. From the analysis, a tendency to increase the resistance to degradation could be seen with the increases in PCL molecular weight (degradation at 315 °C for PEU530, at 325 °C for PEU1000, and at 340 °C for PEU2000). Whereas thermal degradation for the PEUUs was observed at 330 °C for PEUU530, 350 °C for PEUU1000, and PEUU2000 at 370 °C, which indicates that the PEUUs were more stable than PEUs, which could be related to the greater molecular weight of these polymers or the asymmetric structure. In some other polyurethanes or poly(urea-urethane)s, there is a two-step degradation where the urethane or urea groups first degraded and the soft segments degraded at higher temperatures [25]. However, degradation in these polyurethanes took place in a single step, either because both segments degraded at the same time, or because they degraded consecutively without a plateau between both degradations.

**Figure 3:**
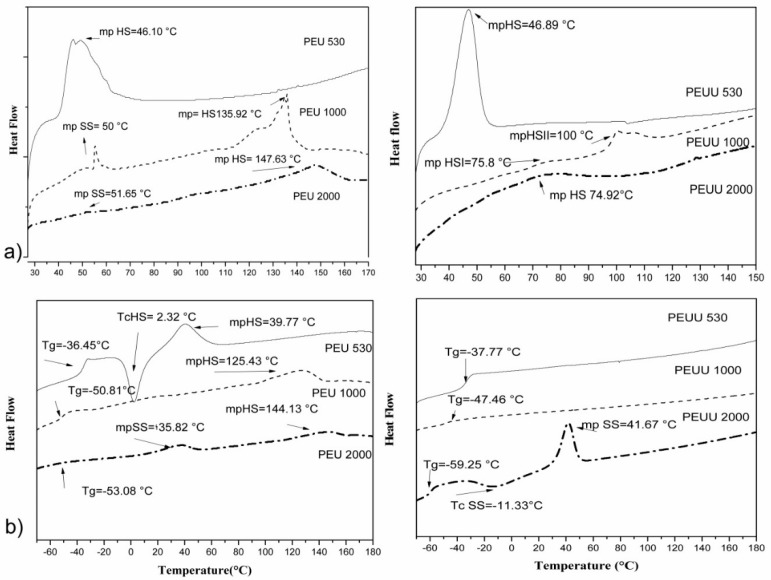
(**a**) First DSC run for the PEUs (left) and PEUUs (right), and (**b**) second DSC run for PEUs (left) and PEUUs (right).

The DSC curves are shown in Figure 3 and summarized in Table 2. The first heating run for PEU530 showed an endothermic peak at 46.10 °C, assigned to the melting of hard segments (HDI-BD). With the low molecular weight obtained, the melting point of the PCL segments would be at low temperatures. Therefore, the endotherm peak cannot be due to the PCL soft segments and can only be due to ordered hard segments. In the second heating run, the *T*_g_ of the material was found at −36.45 °C, followed by an exothermic transition at 2.3 °C and an endothermic transition at 39.8 °C. The *T*_g_ was due to the amorphous material, whereas the exothermic and endothermic transitions were assigned to the crystallization and melting of hard segments. The poly(ester-urethane)s PEU1000 and PEU2000 showed two endothermic transitions in the first heating run, at 50 °C and at 135.9 °C for PEU1000, and at 51.6 °C and at 147.6 °C for PEU2000. The first endotherm peak was related to the crystalline PCL and the second endotherm peak to the crystalline hard segment. In the second heating run, a *T*_g_ at low temperature (below −50 °C) was found due to the amorphous part of the material, followed by an endothermic peak at high temperature (125.4 °C) for PEU1000 due to the melting of the crystalline hard segments, and two endothermic peaks at 35.8 °C and at 144.1 °C for PEU2000 due to the melting of the crystalline soft segments and hard segments, respectively. The existence of separate thermal transitions related to the soft and hard segments demonstrated the existence of phase segregated morphology in these PEU materials. In all cases, the melting points found in the second heat run were lower than those observed in the first heating run, and that could be explained by the fact that crystallinity takes time to be recovered [25].

From the data, it can be seen that when the PCL *M*_w_ was increased, the *T*_g_ value of the amorphous part of the material decreased, showing that the phase separation between soft and hard segments was improved. The *T*_g_ value for PEU530 was significantly higher than for PEU1000 and PEU2000, thus the chain mobility was reduced for this polymer due to the very short chains and to a higher degree of mixing of the segments. The higher degree of mixing of the hard segments with the soft segments in the PEU530 was due to the short length of the hard segments. If the degree of polymerization (*X_n_*) of the hard segments is calculated by using the Carothers equation, it was found that the value of PEU530 was 1.40, which is an average of 0.7 repeating units –(HDI-BD)_n_–. It is accepted that, in general, for linear polyurethanes, a minimum molar ratio of 1:2:1 is necessary in order to achieve a phase-separated morphology. For PEU530, the molar ratio was below this ratio and therefore mixing of the segments was expected to be extensive. For PEU1000 and PEU2000, the molar ratio was above 1:2:1 and a phase-separated morphology was easier to achieve.

The soft segment was unable to crystallize for PEU530, and for PEU1000 and PEU2000, a very low crystallinity in the first heating run was observed. In the second heating run, PEU1000 crystallinity was lost and the crystallinity for PEU2000 was very low. When the PCL chains were introduced in the segmented polyurethane structure, the restrictions in the chain movement due to the anchoring of the PCL ends to the phase-separated hard segments, hampered the crystallization of the chain and reduced or completely suppressed PCL crystallization. Similar behaviors were observed for other polyurethanes based on PCLs of 1000 to 2000 g·mol^−1^ in molecular weight [25]. When the PCL length was increased, the melting point of the hard segments increased as a consequence of the increase in the length of the hard segments. As calculated by using the Carothers equation *X_n_* was 1.40 for PEU530, 3.28 for PEU1000, and 6.30 for PEU2000. Longer hard segments led to a more ordered hard segment phase and higher melting points.

The thermal behavior for the PEEUs was significantly different from the PEUs. In the first heating run, no thermal transitions for the soft segment were found and all of the thermal transitions were assigned to the hard segment. In the second heating run, the endotherm transitions related to the hard segment did not reappear. For PEUU530 and PEUU1000, a single *T*_g_ was found, thus the materials were completely amorphous. For PEUU2000, after the *T*_g_, part of the soft segment crystallized, followed by the melting of the formed crystallites. These results could be interpreted in two ways: either the hard segments mixed with the soft segments after the first heating run, giving a single phase; or the material presented a phase-separated morphology with enriched domains of soft segments and others enriched with hard segments. The *T*_g_ values for the PEUUs were similar to those for the PEUs, thus we concluded that the enriched phase of the soft segment was present in PEUUs with a similar purity than the soft domains of the PEUs. The absence of hard segment transitions in the second heating run for the PEUUs could be due to the presence of a second phase composed of the hard segments mixed with soft segments, that should have a *T*_g_ that was not detected. 

**Figure 4:**
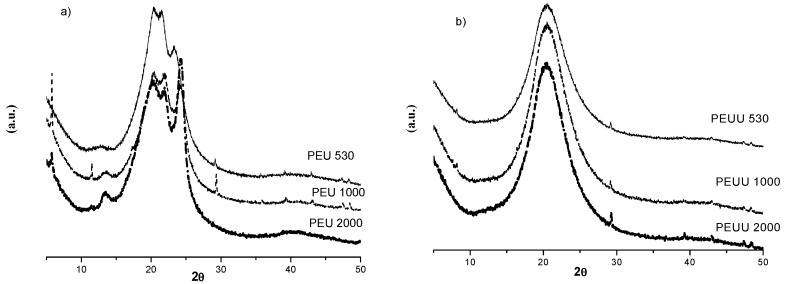
XRD patterns of (**a**) PEUs, and (**b**) PEUUs.

The XRD patterns for the PEUs and PEUUs are shown in Figure 4a,b, respectively. The PEUs were largely amorphous, but some broad peaks between 20° and 25° in 2θ overlapped the amorphous halo. The peak at 25° 2θ increased with the increases of PCL *M*_w_. PCL crystallinity peaks appeared in this range with a maximum at ~21° 2θ. The first heating run in the DSC analysis showed that the PEU530, which showed the same peaks as PEU1000 and PEU2000 in the XRD analysis, did not have PCL crystallinity at room temperature. For this reason, these peaks in the PEUs were assigned to ordered hard segments, where the peak at 25° in 2θ could be related to the hard segments with melting points at higher temperatures.

On the other hand, PEUUs (Figure 4b) showed only a low crystalline peak related to the hard segment crystallinity detected in the first DSC heating run. Due to the asymmetrical structure of the chain extender in the PEUUs, it was expected that the hard segment order was lower than in the PEUs. For this reason, the low crystallinity for PEUUs was detected by DRX.

**Figure 5:**
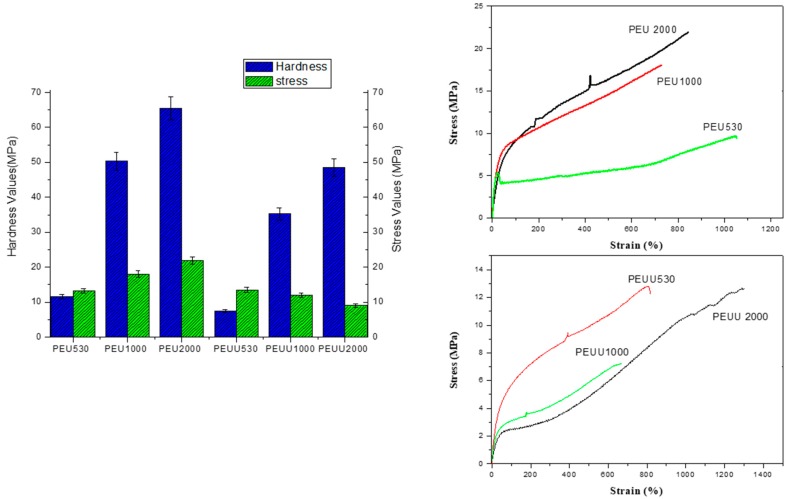
Stress-strain and hardness results of polymers.

The uniaxial stress-strain curves and Vickers hardness results are shown in Figure 5. All polyurethanes behaved as elastomers. PEUs showed stress at break values from 13 to 22 MPa, which increased with the increases in PCL molecular weight and strain at break values above 600%. The shape of the curve showed a fast initial increase in stress when strain reached the yield point (~20%), followed by a slower growth in the stress when strain increased until rupture. The behavior of the curve strain stress was consistent with a phase separated morphology where the hard segment domains act as a reinforcing filler. Hardness values (from 11 to 65 MPa), as the Young Modulus, increased with the increase of PCL molecular weight. The mechanical properties obtained from the PEUUs were lower than for the PEUs, with stress at break values from 13 to 9 MPa and strain at break values above 600%. No trend with the increase in PCL length was found. However, hardness increased from 7 to 48 MPa with increases in PCL length. The greater mechanical properties for PEUs can be explained by the greater phase separation of the segments that implies a higher degree of physical crosslinking and a higher reinforcement effect due to the hard domains.

### 3.2. Biodegradability and Surface Wettability

**Figure 6:**
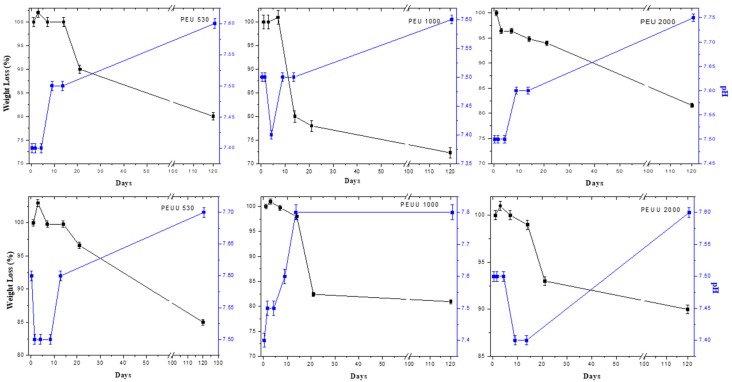
pH and weight changes of polymers submerged in PBS.

The weight and pH changes of the films submerged in PBS are shown in Figure 6. For PEU530 and PEU1000, a swelling was observed at three days; whereas for PEU2000, no swelling was appreciated. On the other hand, pH values remained close to 7.4, but only at 14 days of immersion did the pH values increase, which is related to the beginning of weight loss and the release of amine groups. The total weight loss at 90 days for all PEUs was about 20% wt. The same behavior was observed for the PEUUs with swelling observed at the first three days, the pH values increased at 14 days, and the total weight loss at 90 days was about 15%. The degradation of polyurethanes could be related to the breakage of the ester bonds by the hydrolysis of urethane and urea linkages by enzymatic degradation [29,30]. Thus, the hydrolytic stability was influenced by the change in hard segment structure, which resulted in the PEUUs being more stable in PBS than the PEUs. The higher stability of PEUUs could be related to higher hydrophobicity on the surface. The surface wettability of polyurethanes is controlled by their chemical composition. The contact angle measurements are reported in Table 1. These values were similar to those found in the literature for polyurethanes used in commercial implantable devices (70°–80°) [31].

### 3.3. Cytocompatibility

**Figure 7:**
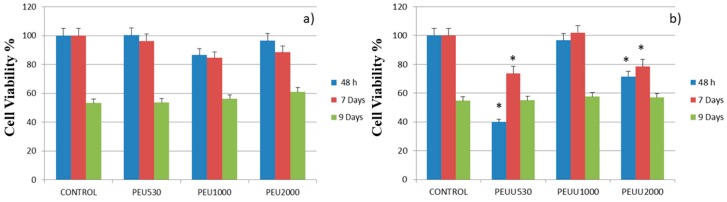
Alamar blue results for (**a**) PEUs and (**b**) PEUUs, using Hob from (INNOPROT, P10979), compared using a one-way ANOVA. * indicates statistical differences from the control *p* < 0.05.

**Figure 8:**
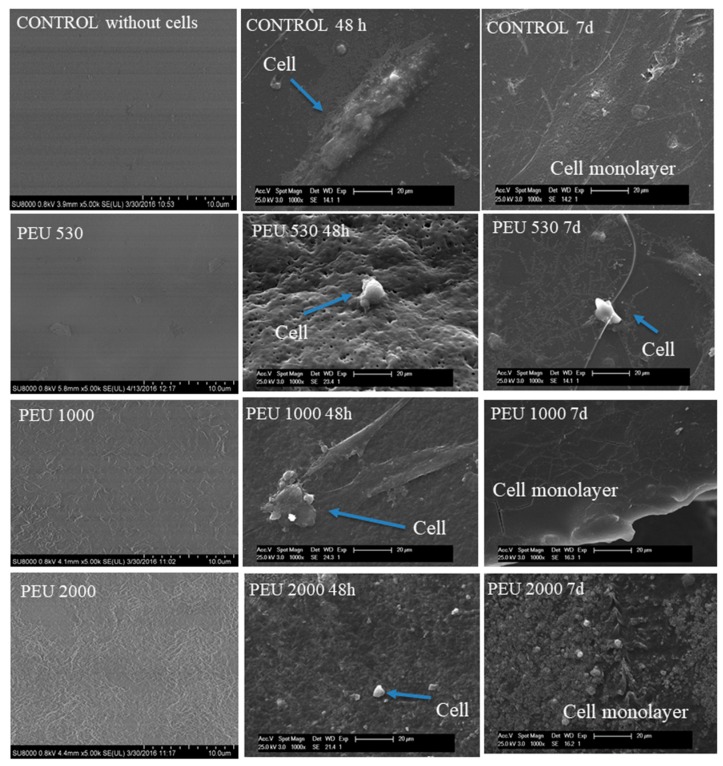
SEM of cell adhesion at 48 h and 7 days on the PEUs.

**Figure 9:**
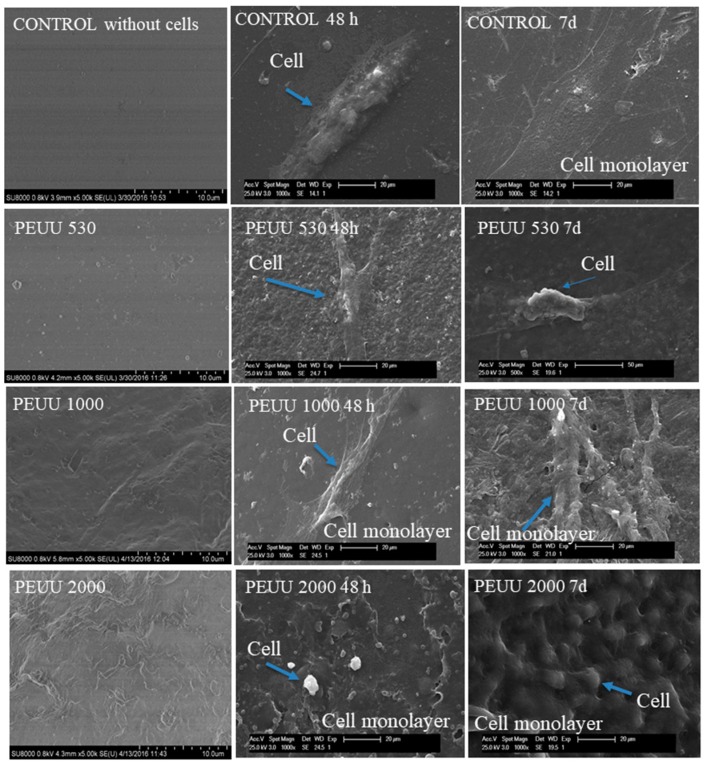
SEM of cell adhesion at 48 h and 7 days on the PEUUs.

The proliferation of the human osteoblastic cell line (Hob) is presented in Figure 7a for PEUs and Figure 7b for PEUUs. For all the PEUs, cell viability was more than 80% at 48 h and seven days of testing. On the other hand, for PEUUs, the cell proliferation grew after 48 h until it reached at least 80% of cell viability at seven days. Finally, at day 9 of testing, the cell viability of all of the polymers and the control sample decreased, which could be related to the high cell confluence reached, with cell viability decreasing as in other in vitro studies [32]. Furthermore, the cell adhesion was visualized by SEM for the PEUs (Figure 8) and PEUUs (Figure 9). As can be seen, for PEU1000 at 48 h, the cells were well expanded on the material surface, whereas for PEU530 and PEU2000, the cells were only deposited in their surface. After seven days, the surface of PEU530 and PEU1000 was fully covered with cells. For PEUUs at 48 h, the cells were deposited on PEUU2000 and well expanded in PEUU530 and PEUU1000. After seven days, all of their surfaces were fully covered with cells, even rising from the cell monolayer that covered the material surface. Similar morphologies for osteoblast like cells seen by SEM were reported [32,33].

## 4. Discussion

Thermoplastic polyurethanes are one of the most versatile elastomeric polymers, their segmented polymeric nature are made up of three components: the bi or multifunctional polyester with a long chain extension (soft segment), the aliphatic diisocianate, and the chain extender, which is usually a diol or a diamine (hard segment). They allow almost limitless potential to control chemical properties and mechanical behavior. For the particular case of bone defect treatments, a flexible soft filler can help to establish intimate contact with surrounding bones to provide a stable bone-material interface for cell proliferation and ingrowth of tissue [34,35].

In this work, the use of diol as the chain extender produced a series of poly-ester-urethanes (PEUs) and the use of an amino acid resulted in a series of poly-ester-urea-urethanes (PEUUs) as can be seen by the FT-IR spectra. The thermal processing window for both series of synthesized polymers serves for applications in tissue engineering, where the temperature required is 37 °C. The thermal behavior of the PEUs showed phase segregation, which was not observed for the PEUUs, related to the different chemical structure of the chain extenders, and the phase segregation was also analyzed by DRX. 

Tensile values for trabecular cancellous bone have been reported to be 5–10 MPa [36,37]. The maximum stress reached values obtained for the PEUs and PEUUs synthesized in this work were above those values, except for PEUU2000, which fell into that range. Since this is the value for the base material for manufacturing scaffolds, a decrease was expected for porous materials [6,38,39]. The better mechanical properties for PEUs were related to the phase separation of the soft and hard segment that involved a higher degree of physical crosslinking. These results are promising for the use of PEUs and PEUUs synthesized from a mechanical point of view. Similar studies of polyurethanes [40] have reported suitable mechanical properties for the same application. On the other hand, the incorporation of nanoparticles as reinforcements [41] or synthesized hybrid materials [42] could improve the mechanical properties, but that was beyond the scope of this manuscript.

There are two ways for the polyurethanes to degrade [29,30], the cracking of the ester bonds by hydrolysis, and the enzymatic degradation of urethane and urea linkages, so the hydrolytic stability is influenced by the change in the nature of the hard segment. The result of the PEUUs being more stable in PBS than PEUs could be related to the major hydrophobicity on the surface observed in the contact angle results. Similar values for polyurethanes used in commercial implantable devices (70–80°) have been reported [31].

Recent studies have reported on biodegradable polyurethanes used as extracellular matrix that supported the attachment and proliferation of osteoblast like cells [37,43,44,45]. The PEUs and PEUUs reached 80% of cell viability at seven days using a human osteoblastic cell line. For PEUs, the cell viability results were better than for the PEUUs, which could be explained by the fact that PEUs are less hydrophobic than PEUUs, and it is known that less hydrophobicity promotes cell proliferation [46]. However, in both series of synthesized polymers, there was good cell proliferation. Furthermore, excellent cell adhesion was observed by SEM, with similar morphologies to the osteoblast like cells reported in the literature [32,33].

## 5. Conclusions

A series of polyester-urethanes and polyester-urea-urethanes were synthesized. Thermal, mechanical, and degradative behavior depend on the *M*_w_ of the soft segment and the nature of the chain extender used. The nature of the chain extender and the *M*_w_ of the soft segment was not affected the cytocompatibility and the adhesion of Hob cells on the surface of the polyurethanes. Both series of polymers show potential to continue with advanced biological studies for bone regeneration.

## Figures and Tables

**Table 1:** Polymers codes, reactant radio used, molecular weights obtained, polydispersity, and contact angle.

PCL *M*_n_	PEU’s Code	Molar Ratio PCL:HDI:BD	PEUs *M*_w_	PEUs *M*_w_/*M*_n_	PEUs Contact Angle	PEUs Code	Molar Ratio PCL:HDI:LYS	PEUUs *M*_w_	PEUUs *M*_w_/*M*_n_	PEUUs Contact Angle
530	PEU 530	1:1.21:0.21	120,800	1.65	67	PEUU 530	1:1.17:0.17	27,600	1.2	70
1000	PEU 1090	1:2.16:1.16	125,300	1.7	70	PEUU 1000	1:1.87:0.87	136,700	1.8	73
2000	PEU 2000	1:3.67:2.67	139,200	1.7	70	PEUU 2000	1:3.01:2.01	196,300	2.0	77

**Table 2:** DSC results of synthesized polymers.

Polymer Codes	First Scan (20–90 °C)		Second SCAN (−80–180 °C)			
	*M*_p_ SS	*M*_p_ HS	*T*_c_	*M*_p_ SS	*M*_p_ HS	*T*_g_
PEU 530	-	46.1	2.3 (HS)	-	39.8	−36.45
PEU 1000	55.3	135.9	-	-	125.4	−50.8
PEU 2000	51.6	147.6	-	35.8	144.1	−53.1
PEUU 530	-	46.9	-	-	-	−33.77
PEUU 1000	-	75.8, 100.1	-	-	-	−47.5
PEUU 2000	-	74.9	11.3 (SS)	41.7	-	−59.2
